# Supplementary material for: Giant magneto-birefringence effect and tuneable colouration of 2D crystal suspensions
Source: Nat Commun. 2020 Jul 24;11:3725. doi: 10.1038/s41467-020-17589-4 (PMC7381639; doi:10.1038/s41467-020-17589-4)
Supplement: Supplementary file 1 — Supplementary Information [file 41467_2020_17589_MOESM1_ESM.pdf]

Supplementary Information

Giant magneto-birefringence effect and tuneable colouration of 2D crystal suspensions

Baofu Ding[1†], Wenjun Kuang[2†], Yikun Pan[1], I. V. Grigorieva[2], A. K. Geim[1,2*], Bilu Liu[1*], Hui-Ming Cheng[1,3,4*]

[1]Tsinghua-Berkeley Shenzhen Institute and Tsinghua Shenzhen International Graduate School, Tsinghua University, Shenzhen 518055, China

[2]Department of Physics and Astronomy, University of Manchester, Manchester M13 9PL, UK

[3]Shenyang National Laboratory for Materials Science, Institute of Metal Research, Chinese Academy of Sciences, Shenyang 110016, China

[4]Advanced Technology Institute, University of Surrey, Guildford, Surrey GU2 7XH, UK

*Corresponding author. Email: hmcheng@sz.tsinghua.edu.cn (H.M.C.); bilu.liu@sz.tsinghua.edu.cn (B.L.); geim@manchester.ac.uk (A.K.G.)

[†]These authors contributed equally to this work.

Supplementary Note 1: Model for magnetically induced birefringence.

We consider a large ensemble of 2D platelets ($10^{11}\sim10^{12}$) suspended in water, where each individual platelet has an anisotropic optical response to linearly polarised light. The birefringence Δn is given by $\Delta n = n_{\parallel} - n_{\perp}$, where $n_{\parallel}$ and $n_{\perp}$ are refractive indices for polarisation parallel and perpendicular to the platelet's surface. In the absence of magnetic field, all 2D Co-TiO$_x$ (CTO) crystals are randomly oriented and exhibit an overall zero birefringence $< \Delta n > = 0$, where $<>$ is the ensemble average. When the magnetic field is applied, the platelets are rotated towards alignment with the field by magnetic torque $\mathbf{\Gamma} = \mathbf{M} \times \mathbf{H}$ due to their finite magnetic anisotropy, $\Delta\chi = \chi_{\parallel} - \chi_{\perp}$. As a result, the system becomes increasingly ordered (anisotropic) and the birefringence builds up with the magnetic field. This can be described using the degree of order in the system, S, so that the birefringence of $< \Delta n >$ is

$$< \Delta n >= \Delta n_{\mathrm{s}} S \tag{1}$$

where Δn_{s} is the saturation birefringence, where all the platelets are fully aligned with the magnetic field. S is a function of the magnetic field and varies between 0 (isotropic) and 1 (all platelets parallel to the field). By analogy with liquid crystal theory[1], we calculate S as

$$S(H) = \frac{3}{2} < \cos^2\vartheta > -\frac{1}{2} \tag{2}$$

where ϑ is the angle between the in-plane director $\mathbf{n}$ of an individual nanoplatelet and the direction of the magnetic field. Since the orientation of suspended platelets by magnetic field is counteracted by Brownian motion, ϑ is subject to Boltzmann statistics, where the probability $\mathrm{d}F$ of finding a platelet at an angle ϑ to $\mathbf{H}$ is related to the energy U associated with its induced magnetisation $\mathbf{M}$ in magnetic field $\mathbf{H}$ as

$$\mathrm{d}F \propto \exp\left(-\frac{U}{k_{\mathrm{B}}T}\right) \sin\vartheta \mathrm{d}\vartheta \tag{3}$$

Here the energy is[1]

$$U = -\int_0^H \mathbf{M} \cdot d\mathbf{H} = -\int_0^H [\chi_{\perp}\mathbf{H} + \Delta\chi(\mathbf{H}\cdot\mathbf{n})\mathbf{n}] \cdot d\mathbf{H} = -\frac{1}{2}\chi_{\perp}H^2 - \frac{1}{2}\Delta\chi H^2 \cos^2\vartheta \tag{4}$$

The term $\frac{1}{2}\chi_{\perp}H^2$ is independent of the platelet's orientation and can be omitted. Then, using (3) and (4), $< \cos^2\vartheta >$ can be calculated as

$$< \cos^2\vartheta >= A^{-1} \int_0^{\pi} \cos^2\vartheta \exp(q\cos^2\vartheta) \sin\vartheta \mathrm{d}\vartheta \equiv L_2(q) \tag{5}$$

where $A = \int_0^{\pi} \exp(q\cos^2\vartheta)\sin\vartheta \mathrm{d}\vartheta$ is the normalization factor, $q = \Delta\chi H^2/2k_{\mathrm{B}}T$, $L_2(q)$ is the generalized 2nd order Langevin function. Combining (1), (2) and (5), we can write the birefringence as

$$< \Delta n >= \Delta n_{\mathrm{s}} S = \frac{\Delta n_{\mathrm{s}}}{2}[3L_2(q) - 1], q = \frac{\Delta\chi H^2}{2k_{\mathrm{B}}T} \tag{6}$$

which establishes a relationship between $< \Delta n >$ and the magnetic field. Note that at small values of q, the generalised 2nd order Langevin function can be approximated as

$$L_2(q) \approx \frac{1}{3} + \frac{4q}{45} \tag{7}$$

Then at small q (low magnetic fields) $< \Delta n > \approx \Delta n_\mathrm{s} \frac{\Delta\chi H^2}{15 k_\mathrm{B} T} \propto H^2$, i.e., the birefringence $< \Delta n >$ varies linearly with H^2, i.e., following the same H dependence as for other systems exhibiting the Cotton-Mouton effect[2,3].

The light intensity transmitted through a birefringent medium sandwiched between two crossed polarisers is

$$I \propto \sin^2 \frac{\delta}{2}, \delta = \frac{2\pi < \Delta n > L}{\lambda} \qquad (8)$$

where δ is the phase retardation between two perpendicular components of linearly polarised light, λ is the wavelength and L the optical path length of the birefringent medium characterised by $< \Delta n >$. The destructive or constructive interference condition is satisfied when the phase difference is

$$\delta = \frac{2\pi < \Delta n(H) > L}{\lambda} = \frac{\pi \Delta n_s L}{\lambda}[3L_2(q) - 1] = (2N-2)\pi \text{ or } (2N-1)\pi, N = 1, 2, 3 \ldots \qquad (9)$$

respectively. Equations (8) and (9) were used to fit the experimental data for the light transmission as a function of magnetic field at $\lambda = 450$ nm (Fig. 4a of the main text). The resulting fit is shown by the solid red line in Fig. 4a, where $\frac{\Delta\chi}{2k_\mathrm{B}T}$ and Δn_s were used as fitting parameters, with the best fit corresponding to $\frac{\Delta\chi}{2k_\mathrm{B}T} \approx 16.0\ \mathrm{T}^{-2}$ and $\Delta n_\mathrm{s} \approx 2 \times 10^{-4}$. At $\mu_0 H = 1$ T and with $\Delta\chi \approx 0.72\chi_\parallel$ obtained from the low-field (linear slope) magnetisation curves $M(H)$ in Supplementary Fig. 4c, this yields the average in-plane magnetisation of an individual platelet $M = \chi_\parallel H \approx 44.4\ k_\mathrm{B} T\ \mathrm{J\,T}^{-1}$. Using this value of M, we can estimate the number of Co^{2+} ions per 2D crystal, N_Co, from the standard expression for induced magnetisation of a paramagnet, $M_\mathrm{platelet} = N_\mathrm{spin/Co} N_\mathrm{Co} J g \mu_\mathrm{B} B_{3/2}(\frac{3\mu_\mathrm{B} H}{k_\mathrm{B} T})$ (see Materials and Methods) and the number of spins per Co atom $N_\mathrm{spin/Co} = 0.6 \pm 0.2$ found from bulk magnetisation measurements using a laminate made of CTO crystals (Supplementary Fig. 4a and see Materials and Methods). Here $J = 3/2$ is the angular momentum quantum number, μ_B the Bohr magneton, $g \approx 2$ the *g*-factor and $B_{3/2}(x)$ the Brillouin function. At room temperature $T = 300$ K and $\mu_0 H = 1$ T, this yields $M \approx (1.5 \pm 0.5) \times 10^{-5}\ N_\mathrm{Co} k_\mathrm{B} T\ \mathrm{J\,T}^{-1}$. Equating this value to M obtained from the fit to experimental optical transmittance ($M = 44.4\ k_\mathrm{B} T\ \mathrm{J\,T}^{-1}$, see above), we obtain $N_\mathrm{Co} \approx (3.5 \pm 1.1) \times 10^6$, corresponding to a number of molecules $Ti_{0.83}Co_{0.1}O_2\ (TBA)_{0.48}$ per platelet $\sim 4 \times 10^7$, in good agreement with the value $\sim 4.5 \times 10^7$ that can be obtained from the average platelet dimensions (flake size ~ 1.5 µm, thickness ~ 1.5 nm, see main text) assuming a density of $4.5\ \mathrm{g\,cm}^{-3}$.

Supplementary Note 2: Measurements of magnetic anisotropy.

a. Magnetic anisotropy characterisation by magnetisation. To determine the magnetic anisotropy of 2D CTO crystals from measurements of magnetisation *M*, exfoliated 2D crystals from a suspension were deposited on a piece of filter paper using vacuum filtration. This produced a ~ 1 µm thick laminate consisting of CTO crystals arranged in approximately parallel layers (Supplementary Fig. 4b). A 3 mm × 3 mm piece of the laminate was then fixed inside a gelatin capsule and oriented in the magnetometer either parallel or perpendicular to the applied magnetic field. The resulting $M(H)$ curves are shown in Supplementary Fig. 4c. The in-plane magnetisation (H parallel to the surface of the laminate) remains significantly higher than the out-of-plane magnetisation ($H \perp$ laminate) over the entire magnetic field range, yielding a finite magnetic anisotropy of the crystals. The finite difference in M at the highest available magnetic field ($\mu_0 H = 7$ T) is presumably due to the fact that no saturation magnetisation is reached either in parallel or perpendicular H. The average of parallel

and perpendicular magnetisation is consistent with the magnetisation of the powder sample containing randomly oriented CTO monolayers (red and blue symbols in Supplementary Fig. 4c).

b. Magnetic anisotropy characterisation by optical measurements. Here, a 650 nm monochromatic light was used as an incident light source. A magnetic field (0 to 900 mT) supplied by a home-made electromagnet was applied to the sample in a direction either parallel or perpendicular to the light path. The transmitted light power was recorded by a power meter (PM200, Thorlabs Inc.) coupled to a photodiode power sensor (S120VC, Thorlabs Inc.). To subtract the background, a reference transmitted intensity I_0 was first recorded without the sample, in zero field and at $\mu_0 H = 800\ \mathrm{mT}$ applied in two different directions (perpendicular and parallel to the light path). Then the transmitted light was recorded with the sample present, under the same field used in the reference measurements. The transmittance was compared as the ratio I/I_0 for the two field directions. As clear from Fig. 2 in the main text, in the presence of the magnetic field ($\mu_0 H = 800\ \mathrm{mT}$), the transmittance is higher in the direction parallel to the field (view from *z*), while no difference is observed when no field is applied. These results are in agreement with expectations for parallel alignment of CTO crystals in the suspension in a finite magnetic field as it permits more light through when the aligned flakes are in-line with the light path. On the other hand, when the aligned flakes are perpendicular to the light path, they block/scatter more light, somewhat similar to window blinds.

Supplementary Note 3: Simulations of transmittance mapping.

As demonstrated in Supplementary Fig. 5b, the normalised optical phase $\delta_{\mathrm{n}}(H)$ for different wavelengths λ shows the same dependence on the magnetic field H, which means that the phase is independently changed by H and λ. This also applies to the birefringence, as $\Delta n(H,\lambda) \propto \delta(H,\lambda)$. Therefore, $\Delta n(H,\lambda)$ can be written as a product of two independent functions of H and λ: $\Delta n(H,\lambda) = g(H)f(\lambda)$. The field dependence is given by (6), $g(H) \propto \frac{3}{2}L_2\left(\frac{\Delta\chi H^2}{2k_{\mathrm{B}}T}\right) - \frac{1}{2}$ and the dispersion of birefringence can be expressed as $f(\lambda) \propto \frac{\lambda^2\lambda^{*2}}{\lambda^2-\lambda^{*2}}$, where λ^* is the so-called resonant wavelength[4]. In our case $\lambda^* = 251.3\ \mathrm{nm}$ was found from the fit to the experimental dependence of Δn on λ (Fig. 4b in the main text). It follows that

$$\Delta n(H,\lambda) = A_0\left[\frac{3}{2}L_2\left(\frac{\Delta\chi H^2}{2k_{\mathrm{B}}T}\right) - \frac{1}{2}\right]\left(\frac{\lambda^2\lambda^{*2}}{\lambda^2 - \lambda^{*2}}\right) \tag{10}$$

where A_0 is a numerical factor. Substituting equation (10) in $I \propto \sin^2\frac{\pi\Delta n(H,\lambda)L}{\lambda}$ and using $\frac{\Delta\chi}{2k_{\mathrm{B}}T} = 16.0\ \mathrm{T}^{-2}$ (see above), we obtained the simulated pattern of transmitted intensity shown in Fig. 3c in the main text ($A_0 = 2.04 \times 10^{-9}\ \mathrm{nm}^{-2}$).

Supplementary Note 4: Criterion for the magneto-colouration in birefringent system.

To achieve the magneto-chromatic effect in a birefringent system, the magnetic field-induced shift of the maximum light transmission (corresponding to constructive interference) should be sufficiently different for different wavelengths (as in Fig. 3b in the main text). In transmitted intensity maps such as shown in Fig. 3a, this condition can be interpreted as a not too large slope of high-intensity stripes, to allow discrimination of different colours. Mathematically, this condition can be expressed as

$$\Delta H \times \frac{d\lambda_{\mathrm{c}}}{dH} < \Delta\lambda_{\mathrm{v}} \tag{11}$$

over the visible wavelength range, where ΔH is the width of the Nth order of the high intensity transmission stripe, λ_{c} the constructive interference wavelength (see main text), $\frac{d\lambda_{\mathrm{c}}}{dH}$ is the slope of the stripe and $\Delta\lambda_{\mathrm{v}}{\sim}340\ nm$ is the wavelength range for the visible light. As shown in the previous section, the birefringence is independently changed by the field and the wavelength, $\Delta n = g(H)f(\lambda)$. Using the constructive interference condition $\delta = \frac{2\pi\Delta nL}{\lambda_{\mathrm{c}}} = (2N-1)\pi$, we can find the slope $\frac{d\lambda_{\mathrm{c}}}{dH}{\sim}\frac{2L}{2N-1}\frac{dg(H)}{dH}f(\lambda)$. On the other hand, the width of the high-intensity stripe, ΔH, can be defined as the field range over which the light intensity changes from maximum (constructive interference) to minimum (destructive interference) for the Nth order, $\Delta H{\sim}\lambda/[2L\frac{dg(H)}{dH}f(\lambda)]$. Combining the two expressions gives $\Delta H \times \frac{d\lambda_{\mathrm{c}}}{dH}{\sim}\frac{\lambda}{2N-1}$. It is clear that condition (11) cannot be satisfied for $N = 1$ (1st order constructive interference) where $\Delta H \times \frac{d\lambda_{\mathrm{c}}}{dH}{\sim}\lambda > \Delta\lambda_{\mathrm{v}}{\sim}340$ nm for any value of λ in the visible wavelength range. However, it can be readily met for $N = 2$ where $\Delta H \times \frac{d\lambda_{\mathrm{c}}}{dH}{\sim}\frac{\lambda}{3} < 340\ \mathrm{nm}$ for all visible wavelengths, and for all $N > 2$. The above analysis allows us to conclude that, to achieve magneto-colouration effect, the phase retardation δ should satisfy the condition $\delta \geq 3\pi$.

Supplementary Table 1:

Comparison of the Cotton-Mouton coefficients for our 2D CTO suspension and typical organic liquid crystals, liquids, gases and ferrofluids

Materials	Cotton-Mouton Coefficient ($T^{-2}m^{-1}$)	λ (nm)	Reference
2D Co-TiO_x (CTO)	1.4×10^3	450	This work
Organic liquid crystal			
*n*CB (*n*=5~12)	0.1-3.0	632.8	5
*n*OCB (*n*=5~11)	0.2~5.1	632.8	5
PCH*n* (*n*=3,4,5,7)	0.1~0.2	632.8	5
MBBA	3.3	632.8	5
EBBA	1.5	632.8	5
4-O6	0.9	632.8	5
4-O7	1.1	632.8	5
Liquid			
Isonicotinic acid	2.4×10^{-2}	515	3
Benzene	6.9×10^{-3}	515	3
Acetone	5.3×10^{-4}	515	3
Carbon disulfide	5.9×10^{-3}	515	3
Acetonitrile	7.0×10^{-7}	632.8	6
Biological material			
Ferritin	4.0×10^{-2}	632.8	7
Fe-sucrose	1.3	632.8	8
Fe-polymaltose	0.2	632.8	8
Fe-dextran	0.4	632.8	8
Fe-dextran	4.0×10^{-3}	632.8	8
Iron based ferrofluid			
Magnetite	2.3×10^3	632.8	7
Gas			
Helium	2.4×10^{-10}	1064	9
Neon	1.3×10^{-9}	790	10
Argon	2.6×10^{-8}	514.5	10
Xenon	2.2×10^{-8}	514.5	11

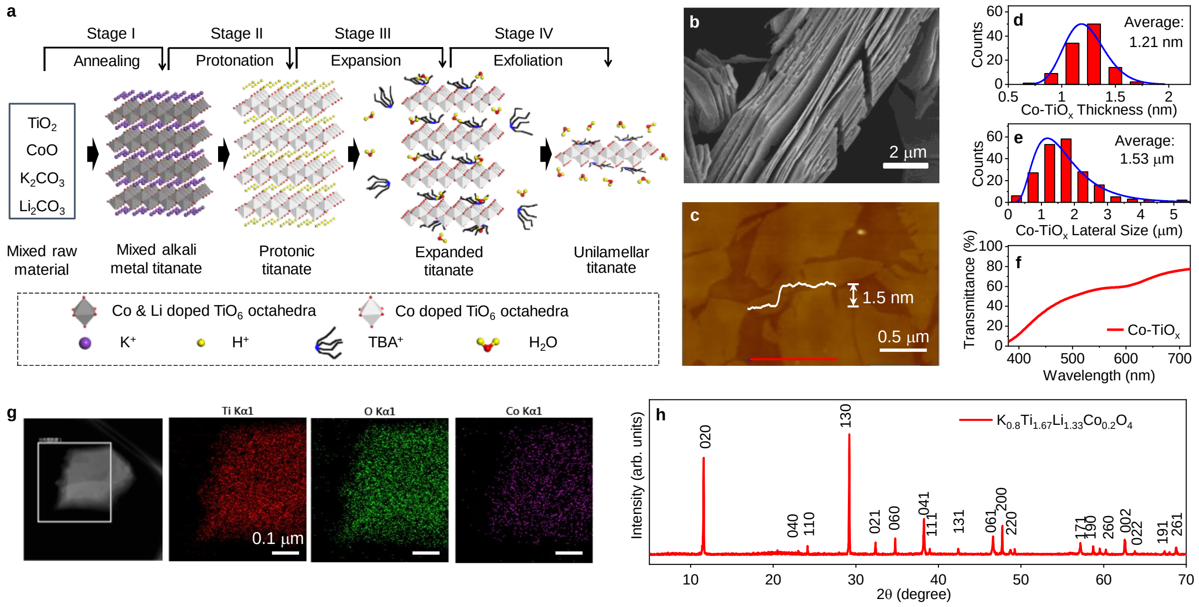

Supplementary Fig. 1| Synthesis and characterisation of 2D CTO. **a**, Schematic of CTO synthesis using a four-stage approach described in Materials and Methods. **b,** Scanning electron microscope (SEM) image of the stage-II product showing a layered structure with expanded interlayer spacing due to proton exchange with alkali ions. **c-e,** Thickness and lateral size of the stage-IV product characterised by atomic force microscopy (AFM). The exfoliated CTO crystals show an average thickness of 1.2 nm (1-2 layers) and an average lateral size of 1.5 μm. **f**, Optical transmittance of non-polarised light through the 0.02 vol% aqueous suspension of 2D CTO crystals in 10 mm x 10 mm cuvette in the visible range. **g**, Energy-dispersive X-ray spectroscopy (EDX) mapping of the elemental composition of our 2D CTO. The sample shows uniform Co doping with an atomic ratio between the elements Co:Ti:O = 0.2:1.65:4. **h**, X-ray diffraction (XRD) pattern of the stage-I product.

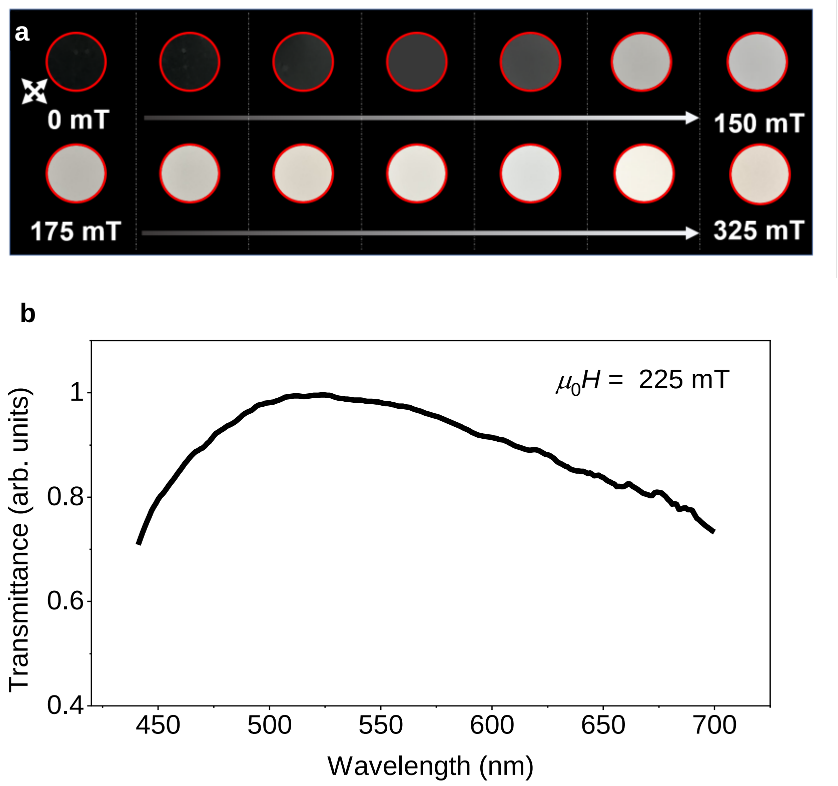

Supplementary Fig. 2| Transmission of white light through the CTO suspension in low magnetic fields. a, Optical images of the transmitted light as the field increases from 0 to $\mu_0 H$ = 325 mT. **b**, Normalised spectra at $\mu_0 H$ = 225 mT showing high transmission over the whole visible range.

a

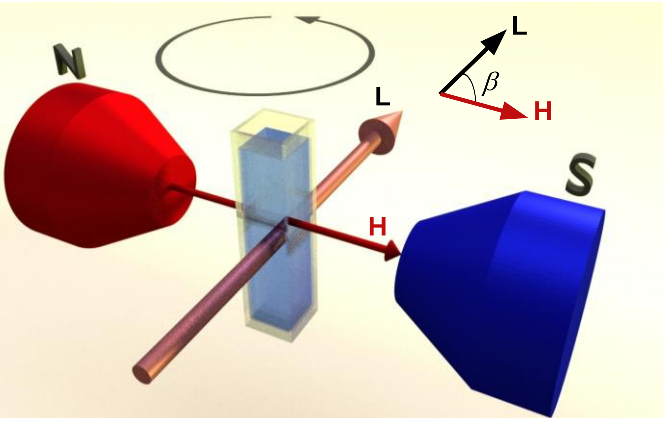

b

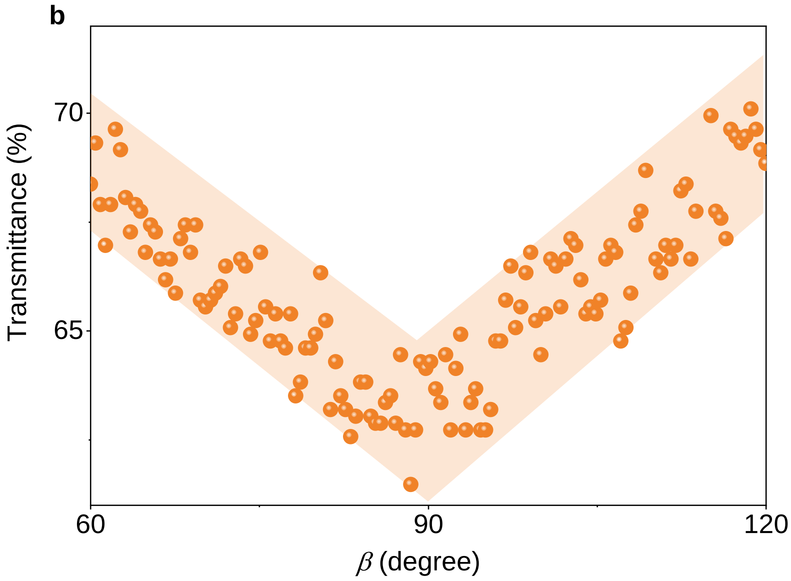

Supplementary Fig. 3| Angle dependence of transmittance. **a**, Schematic of our experimental setup for measuring the optical transmission through the 2D CTO suspension using a 500 mT permanent magnet. β denotes the angle between the incident light direction and magnetic field. **b**, Dependence of the light transmission on β showing a minimum for $\beta = 90°$ (view from *x* in Fig. 2b). The symbols represent experimental data, and the shade areas are a guide to the eye. The angle is restricted to ±30° around the perpendicular configuration, beyond which the light gets blocked by the magnet.

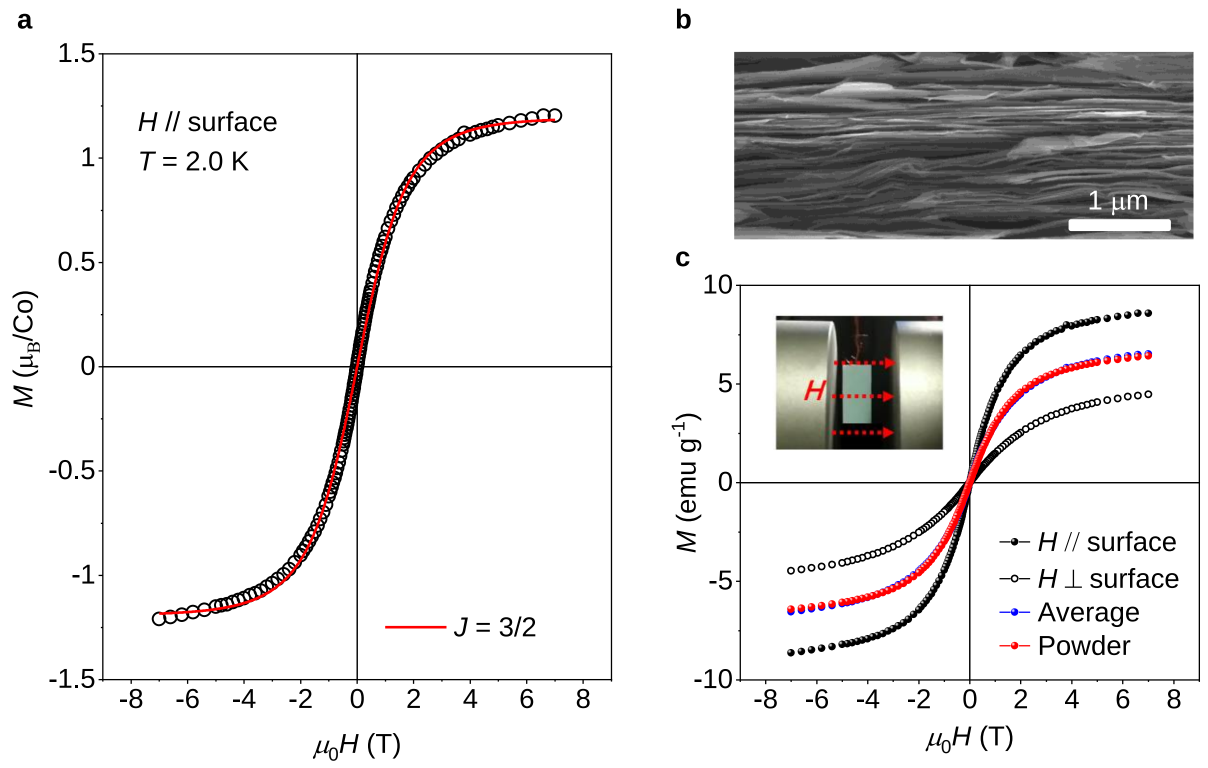

Supplementary Fig. 4| Magnetic response of CTO crystals. **a**, Magnetisation $M(H)$ of CTO powder at 2 K shows a typical paramagnetic response with no hysteresis. Red solid line is the best fit to the Brillouin function $M = N_{\mathrm{spin/Co}} gJ\mu_{\mathrm{B}} B_J(x)$ corresponding to the angular momentum $J = 3/2$, consistent with the expected spin state of Co atoms. The number of spins found per doped Co atom is $N_{\mathrm{spin/Co}} = 0.6 \pm 0.2$. Details of the fitting are given in Supplementary Notes 1 and 3. The relatively large error in $N_{\mathrm{spin/Co}}$ is due to the very small mass of the laminate, $m = 0.20 \pm 0.15$ mg, and the associated error in calculating mass magnetisation. **b**, Cross-sectional SEM image of the CTO laminate used for measurements of magnetic anisotropy shown in (c). Parallel arrangement of the individual crystals in the laminate is clearly seen. **c**, Magnetisation of the CTO laminate measured in the magnetic field parallel (filled black symbols) and perpendicular (open black symbols) to the sample surface. The blue symbols show the average for the in-plane and out-of-plane magnetisations. The red symbols are for magnetisation of the powder, in which CTO crystals are randomly oriented with respect to the field. Inset: optical image of a rectangular sample of the CTO laminate suspended by a string between the poles of an electromagnet. The laminate sample aligns parallel to the field ($\mu_0 H = 0.6$ T).

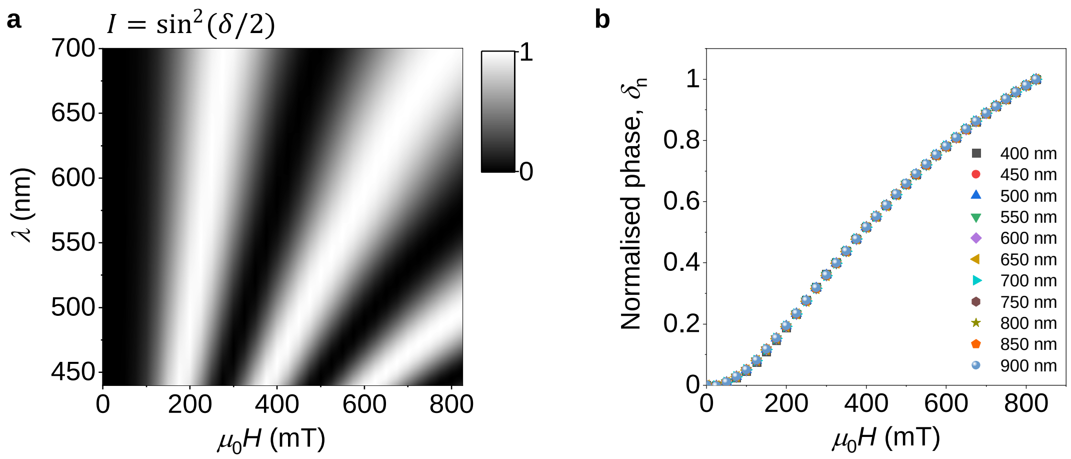

Supplementary Fig. 5| Phase retardation measurements. **a**, Map of transmitted intensity calculated as by $I = \sin^2(\delta/2)$ from the phase retardation $\delta(H, \lambda)$ measured directly using a spectroscopic ellipsometer. The intensity map accurately reproduces the measured field-dependent transmission spectra shown in Fig. 3a in the main text, providing further evidence that the observed magneto-chromatic effect arises from the field-induced birefringence of the CTO suspension. **b**, Dependence of the normalised phase retardation $\delta_n = \delta(H)/\delta_{max}$ on H^2 at different wavelengths, see legend. The lack of wavelength dependence (data for different λ overlap) suggests that the birefringence is independently linked to the magnetic field and the optical wavelength.

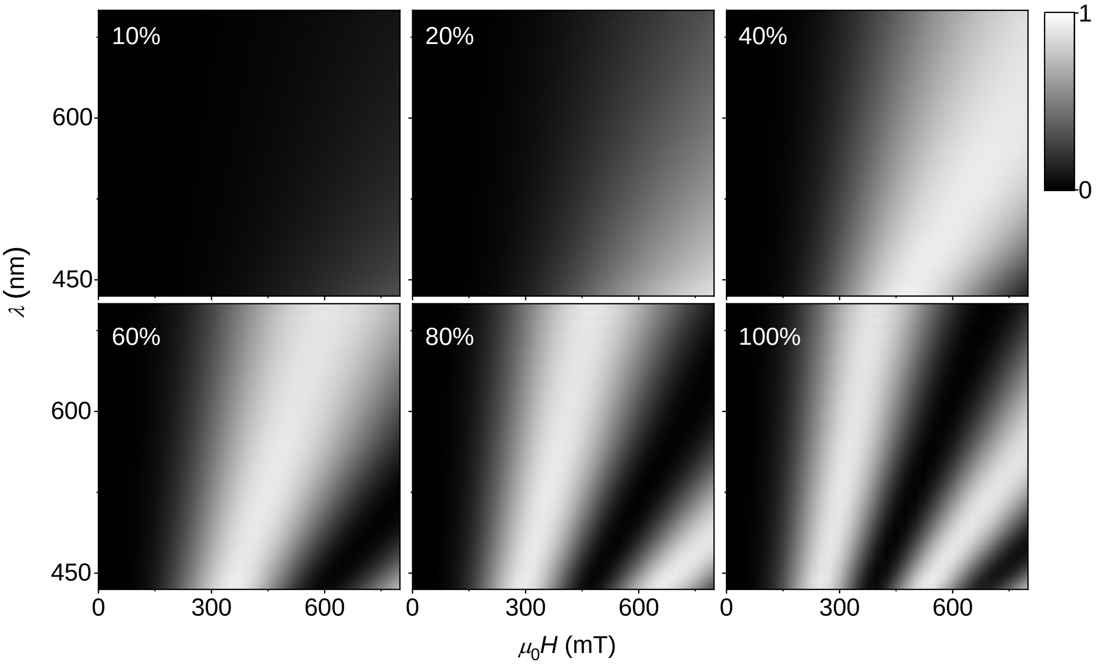

Supplementary Fig. 6| Effect of 2D CTO concentration on the magneto-optical spectra. The concentration used in most of our measurements (suspension with 0.02 vol% fraction of 2D CTO in water) is taken as 100%. All the other concentrations were prepared by diluting the original suspension with water.

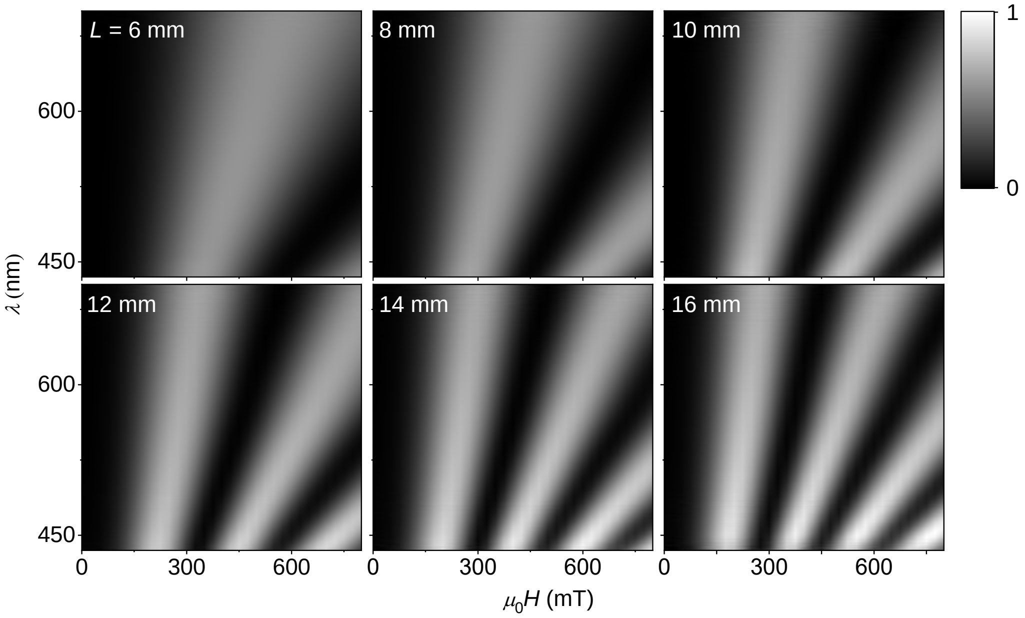

Supplementary Fig. 7| Effect of the optical path on magneto-optical spectra. The labels indicate the optical path length (length of the cuvette along the light path). Higher order constructive/destructive interference fringes appear for long optical lengths.

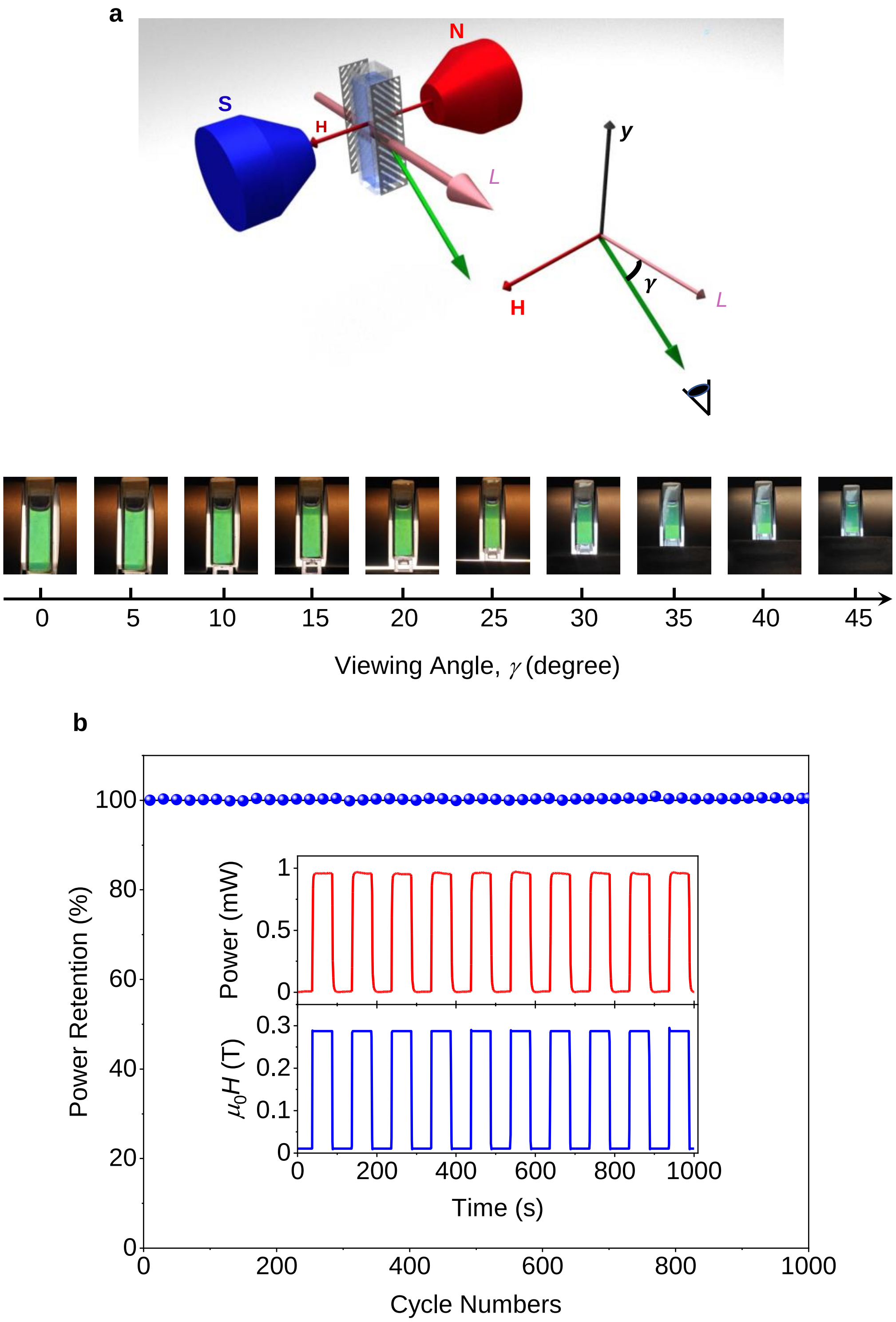

Supplementary Fig. 8| Viewing angle and stability of our CTO suspension. a, Photographs show transmitted colours of the suspension in 700 mT for viewing angles from 0° to 45°. The top panel is a schematic of our setup: the viewing angle (γ) was varied in the plane of the incident light (L) and perpendicular to the field **H**. **b**, Cycling test for the CTO suspension. The main figure shows the retention of the transmitted power (normalised to its initial state). Insets: time-dependent transmitted power (upper panel) as the field (290 mT) is turned on and off every 100 s (lower panel).

a

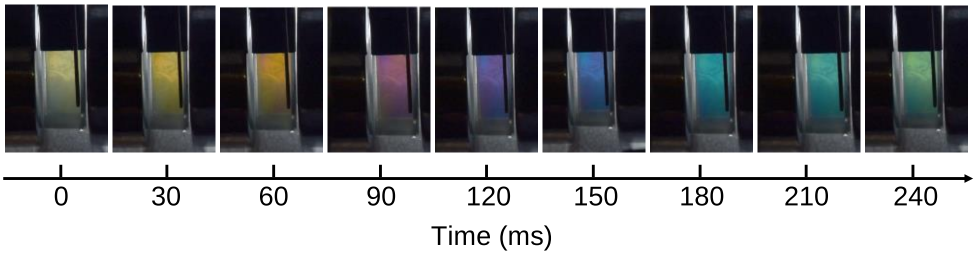

b

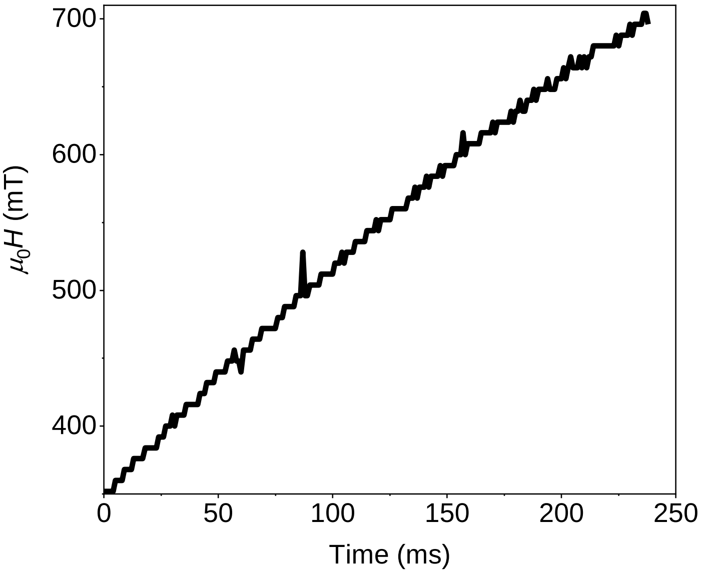

Supplementary Fig. 9| Time response of the magneto-chromatic effect. a, Snapshots of the CTO suspension taken with every 30 ms as the field was increased from 350 mT to 700 mT. **b**, Changes in the magnetic field during the recorded sequence of the images in **a**.

Supplementary References

1 Stephen, M. J. & Straley, J. P. Physics of liquid crystals. *Rev. Mod. Phys.* **46**, 617-704 (1974).

2 Rizzo, C., Rizzo, A. & Bishop, D. M. The Cotton-Mouton effect in gases: Experiment and theory. *Int. Rev. Phys. Chem.* **16**, 81-111 (1997).

3 Wilson, S. R., Ridler, P. J. & Jennings, B. R. A simple apparatus for the measurement of the Cotton-Mouton effect in particulate suspensions. *IEEE Trans. Magn.* **33**, 4349-4358 (1997).

4 Wu, S.-T. Birefringence dispersions of liquid crystals. *Phys. Rev. A* **33**, 1270-1274 (1986).

5 Blachnik, N., Kneppe, H. & Schneider, F. Cotton-Mouton constants and pretransitional phenomena in the isotropic phase of liquid crystals. *Liq. Cryst.* **27**, 1219-1227 (2000).

6 Fahleson, T., Olsen, J. M. H., Norman, P. & Rizzo, A. A QM/MM and QM/QM/MM study of Kerr, Cotton–Mouton and Jones linear birefringences in liquid acetonitrile. *Phys. Chem. Chem. Phys.* **20**, 3831-3840 (2018).

7 Koralewski, M. *et al.* Magnetic birefringence of natural and synthetic ferritin. *J. Magn. Magn. Mater.* **323**, 2413-2417 (2011).

8 Koralewski, M., Pochylski, M. & Gierszewski, J. Magnetic properties of ferritin and akaganeite nanoparticles in aqueous suspension. *J. Nanopart. Res.* **15**, 1902 (2013).

9 Cadène, A. *et al.* Faraday and Cotton-Mouton effects of helium at λ = 1064 nm. *Phys. Rev. A* **88**, 043815 (2013).

10 Muroo, K., Ninomiya, N., Yoshino, M. & Takubo, Y. Measurement of the Cotton–Mouton constants of noble atoms. *J. Opt. Soc. Am. B* **20**, 2249-2254 (2003).

11 Cadène, A. *et al.* Circular and linear magnetic birefringences in xenon at λ = 1064 nm. *J. Chem. Phys.* **142**, 124313 (2015).
