# Supplementary material for: Giant magneto-birefringence effect and tuneable colouration of 2D crystal suspensions
Source: Nat Commun. 2020 Jul 24;11:3725. doi: 10.1038/s41467-020-17589-4 (PMC7381639; doi:10.1038/s41467-020-17589-4)
Supplement: Supplementary file 3 — Description of Additional Supplementary Files [file 41467_2020_17589_MOESM3_ESM.pdf]

Description of Additional Supplementary Files

File name: Supplementary Movie 1

Description: Magnetic-field tuneable colouration of 2D cobalt-doped titanium oxide (CTO) suspensions. The CTO suspension placed in a uniform magnetic-field region between two electromagnet poles shows a uniform colour in the presence of crossed polarisers. The first and second demos illustrate that when the CTO suspension passes through a gradient field region, a corresponding rainbow of colours are seen. The one-to-one colour correspondence with the magnetic field is presented in the third demo.
